# The interactome of *Streptococcus pneumoniae* and its bacteriophages show highly specific patterns of interactions among bacteria and their phages

**DOI:** 10.1038/srep24597

**Published:** 2016-04-22

**Authors:** Rachelle Mariano, Stefan Wuchty, Maria G. Vizoso-Pinto, Roman Häuser, Peter Uetz

**Affiliations:** 1 Dept. of Computer Science, University of Miami, Coral Gables, FL 33146, USA; 2 Center for Computational Science, University of Miami, Coral Gables, FL 33146, USA; 3 Max von Pettenkofer-Institute, Department of Virology, Ludwig-Maximilians-University, Munich, Germany; 4 Instituto Superior de Investigaciones Biológicas (INSIBIO), CONICET-UNT, and Instituto de Fisiología, Facultad de Medicina, UNT. San Miguel de Tucumán, Argentina; 5 Genomics and Proteomics Core Facilities, German Cancer Research Center, Im Neuenheimer Feld 580, 69120 Heidelberg, Germany; 6 Center for the Study of Biological Complexity, Virginia Commonwealth University, Richmond, VA 23284, USA

## Abstract

Although an abundance of bacteriophages exists, little is known about interactions between their proteins and those of their bacterial hosts. Here, we experimentally determined the phage-host interactomes of the phages Dp-1 and Cp-1 and their underlying protein interaction network in the host *Streptococcus pneumoniae.* We compared our results to the interaction patterns of *E. coli* phages lambda and T7. Dp-1 and Cp-1 target highly connected host proteins, occupy central network positions, and reach many protein clusters through the interactions of their targets. In turn, lambda and T7 targets cluster to conserved and essential proteins in *E. coli*, while such patterns were largely absent in *S. pneumoniae*. Furthermore, targets in *E. coli* were mutually strongly intertwined, while targets of Dp-1 and Cp-1 were strongly connected through essential and orthologous proteins in their immediate network vicinity. In both phage-host systems, the impact of phages on their protein targets appears to extend from their network neighbors, since proteins that interact with phage targets were located in central network positions, have a strong topologically disruptive effect and touch complexes with high functional heterogeneity. Such observations suggest that the phages, biological impact is accomplished through a surprisingly limited topological reach of their targets.

Protein-protein interaction networks (PINs) have become a key measure of cellular organization^1^. Surprisingly, only few networks have been elucidated to date, and most suffer from being incomplete. Although tens of thousands of completely sequenced genomes exist, less than a dozen bacterial interactomes have been tackled^2^,^3^,^4^,^5^,^6^,^7^. While thousands of interactions between human host and human virus proteins have been detected over the last decades, protein interaction interfaces between bacteriophages and hosts have been studied in detail only for a few phages such as lambda and T7 in *E. coli*^8^.

For the first time, we present the interactome of the bacterium *S. pneumoniae* and its interactions with two phages, Dp-1 and Cp-1. Although similar studies have been carried out in human viruses, no such comparisons between bacterial systems have been presented for phages to date. The direct comparison of host-virus interactome data is difficult, since such interactions were determined by different and independent studies by methodologies that detect a different subset of interactions^9^,^10^,^11^. To combat this issue we analyzed the interactomes under identical conditions to provide a uniquely standardized network evaluation.

We have previously investigated the interactomes of both Cp-1 and Dp-1 without considering interactions with their host^12^,^13^. Furthermore, most of Cp-1 and Dp-1’s genes remain poorly characterized. To provide a benchmark for *Streptococcus*-phage interactions, we compiled reference interactome datasets from the well-characterized *E. coli* bacteriophages T7 and lambda. Lambda and T7 differ significantly in their host interaction patterns, reflecting their different biology^8^. T7 is a lytic phage while lambda is lysogenic. In addition, lambda uses an unusually high number of protein modifications such as proteolytic cleavages. As a consequence, T7 and lambda serve as a model for other phages infecting the same host while using different propagationstrategies.

A comparison of the two *E. coli* phages (T7 and lambda) with two phages of *Streptococcus* (Cp-1 and Dp-1) allowed us for the first time to extract general interaction patterns of phages with different bacterial hosts. Notably, we show that these interactions are surprisingly specific for each phage even if we use standardized methods to detect host-virus interactions. Our data demonstrate that each phage has evolved species-specific adaptations that manipulate varied facets of host machinery, reflecting the underlying host-phage coevolution.

## Results

### Interactions among *S. pneumoniae* and its phages

Using a yeast-two hybrid approach, we screened a collection of 1,704 prey clones derived from *S. pneumoniae* with all 28 open reading frames of the Cp-1 genomes as baits. Similarly, all 72 deduced proteins of phage Dp-1 were screened against the same *S. pneumoniae* prey collection (see Methods for details). The Cp-1 screens allowed us to find 11 interactions between 7 phage and 10 host proteins. While the significance of these interactions remains unknown, tail protein N was found to interact with oligoendopeptidase F, possibly indicating proteolytic cleavage of N. Uridine kinase (Cpl1) interacts with lysozyme, a critical enzyme for bacterial lysis, prompting us to test whether the kinase can affect lysozyme function or *vice versa*. Despite the fairly strong interaction (as measured by 3-AT titration) we did not detect an effect of uridine kinase on lysozyme activity or *vice versa*. All other Cp-1-host interactions involved phage proteins of unknown function whose biological role remains uncertain. All interactions that involved proteins of Cp-1 are listed in Table 1.

**Figure 1:**
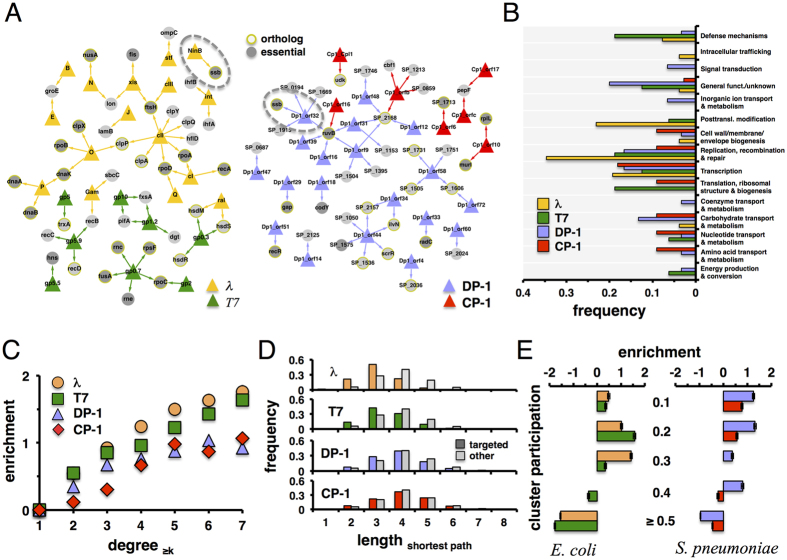
Comparison of the host-phage interaction interface of lambda and T7 with *E. coli* and Dp-1 and Cp-1 with *S. pneumoniae*. In (**A**) we collected 36 protein-protein interactions between 16 lambda and 23 *E. coli* proteins as well as 19 interactions between 8 T7 and 14 *E. coli* proteins from the literature. In turn, we found 11 interactions between 7 Cp-1 and 10 proteins of *S. pneumoniae,* while we determined 38 interactions between 19 Dp-1 and 24 proteins of *S. pneumoniae.* In both host organisms we observed a limited number of proteins that were targeted by lambda and T7 (RecB, HsdM, HsdS) as well as Dp-1 and Cp-1 (RuvB, SP_2168). Furthermore, we observed that targets are frequently essential and have orthologs in the other organism. Notably, *Ssb* is evolutionarily conserved in both *E. coli* and *S. pneumoniae* and is targeted by lambda as well as Dp-1 (dashed circles). In (**B**) we determined the frequency of phage-targeted proteins and their functional classes. (**C**) Utilizing protein interactions in *E. coli* we observed that lambda and T7 targets appear to have an increasing number of interaction partners. Focusing on *S. pneumoniae*, we obtained similar results when we considered targets of bacteriophages Dp-1 and Cp-1. In (**D**) we calculated shortest paths from targeted proteins to all other host proteins in the corresponding host interaction networks of *E. coli* and *S. pneumoniae*. Comparing distributions that correspond to lambda and T7, we found that the lengths of shortest paths from targeted proteins are significantly shorter than paths from non-targeted proteins (Student’s t-test, P < 10^−11^). We obtain a similar result when we considered targets of phages Dp-1 and Cp1 (P < 10^−12^). In (**E**) we calculated the cluster participation coefficient of proteins that were targeted by bacteriophages of *E. coli* and *S. pneumoniae*. As a null model, we randomly sampled such sets of targeted proteins 10,000 times. Determining their enrichment, we observed that targeted proteins appear to predominantly reach into more complexes through their interactions than randomly expected. Error bars indicate 95% confidence intervals. Colors as in (**B**) and (**C**).

With 72 ORFs, phage Dp-1 is considerably larger than Cp-1 (28 ORFs). When we screened the Dp-1 ORFeome against our *S. pneumoniae* prey library we found 38 interactions between phage and host proteins (Table 2). Notably, we observed that RuvB was strongly targeted by the highest number of phage host proteins, indicating that the phage interferes with DNA repair and recombination functions. Furthermore, this protein also weakly interacts with a hypothetical protein of Cp-1 (Fig. 1A).

All interactions were verified using a LuMPIS assay (Tables 1 and 2), confirming 35 out of the 38 Dp-1 and 8 out of the 11 Cp-1 interactions when we used a cut-off of ≥3 LIR units even though a total of 12 PPIs were borderline positive at LIR values of ≈3 (see Materials and Methods for details). Note, however, that we used all Y2H interactions for the network analysis described below, given that this cut-off is somewhat arbitrary.

### Phage-host interactions in bacteria are highly species-specific

As a well-investigated benchmark of host-bacteriophage interfaces, we comprehensively surveyed the literature and curated 36 interactions between 16 lambda and 23 *E. coli* proteins in *E. coli*^8^. Similarly, we compiled 19 interactions between 8 T7 and 14 *E. coli* proteins^8^. Mapping such interactions (Fig. 1A), we observed that the majority of host proteins are targeted by one phage protein, while overlaps of phage-specific sets of targeted proteins are limited in both host organisms. Furthermore, we found that essential proteins appeared enriched in such interaction interfaces. While numerous targeted proteins had orthologs in the opposite organism, *Ssb* was the only evolutionarily conserved protein that was targeted in both host bacteria (Fig. 1A). In Fig. 1B we grouped targeted protein sets according to broad functional classes that were defined by clusters of orthologous groups (COGs)^15^,^16^. Determining the occurrence of functions in the host-phage interfaces of both organisms, we found that targeted proteins mostly carried transcription, replication, recombination, and repair functions.

To investigate the location of targeted proteins in *E. coli*, we assembled a network of 11,463 interactions between 2,765 proteins^2^,^17^,^18^. As for *S. pneumoniae*, we experimentally determined a network of 2,036 interactions between 836 proteins using a two-hybrid approach (see Methods for details). Furthermore, we accounted for 197 interactions that were previously determined by a microfluidic high-throughput assay^19^. Specifically, we calculated the enrichment of targeted proteins as a function of their degree (Fig. 1C), suggesting that host proteins with an increased number of interaction partners are prime targets for phages in *E. coli*. We found a similar, yet weaker trend for genes in *S. pneumoniae* that were targeted by Dp-1 as well as Cp-1 (Fig. 1C). Similar observations have been previously reported for human host-viral^20^,^21^ as well as host-parasite interactions^22^,^23^.

As a corollary to the observed phages’ preference to target central positions in the protein interaction network of *E. coli* and *S. pneumoniae*, we hypothesized that targeted proteins allow the pathogen to reach other proteins efficiently. In particular, we calculated shortest paths from targeted proteins to other proteins in the underlying interaction networks. As for bacteriophages of *E. coli,* we observed that lengths of shortest paths from proteins that are targeted by lambda and T7 respectively were significantly shorter than paths from non-targeted proteins (P < 10^−9^, Student’s t-test) (Fig. 1D). Notably, we found similar results when we considered shortest paths from proteins that were targeted by phages Dp-1 and Cp-1 through an interaction network to other proteins in *S. pneumoniae*.

### Protein complexes are targeted by phages

Protein complexes present another level of cellular organization. To obtain protein clusters in the interaction network of proteins in *S. pneumoniae*, we applied the Markov cluster (MCL) algorithm with varying values of its inflation parameter to modulate the granularity (i.e. size) of clusters. Utilizing COG^15^,^16^ annotations, we calculated the functional coherence (see Methods) of each cluster. Such a measure tends to decrease with large clusters and *vice versa*. To balance such a trend we calculated the modularity efficiency *E*_*M*_ of a given set of clusters^24^. We obtained a maximum value of *E*_*M*_, with inflation parameter of 1.6 in *S. pneumoniae*, providing 148 clusters. As for *E. coli*, we utilized a set of 517 protein complexes from a co-affinity purification study that was followed by mass spectrometry analyses^17^. We calculated a complex participation coefficient of each *E. coli* protein that indicates if a protein mainly interacts with proteins in the same or different complexes. In particular, a protein’s complex participation coefficient tends toward 1 if it predominantly interacts with proteins in the same complex. In turn, such a value tends to 0 if the given protein mainly interacts with proteins in other complexes. Binning proteins according to their corresponding complex participation coefficient, we calculated the fraction of targeted proteins in each group. As a null model we randomly sampled sets of targeted proteins, allowing us to determine the enrichment of targeted proteins as the ratio of observed and expected fractions of targeted proteins in each bin. Figure 1E clearly indicates that *E. coli* proteins that were targeted by bacteriophage lambda and T7, respectively, were enriched in groups of proteins that reached numerous complexes. Calculating their enrichment in bins of increasing complex participation, we confirmed our initial observation that proteins in *S. pneumoniae* targeted by phages Dp-1 and Cp-1 mostly connect different clusters through their interactions (Fig. 1E).

### Phage proteins target clusters of host proteins as well as, essential and orthologous proteins in bacteria

**Figure 2:**
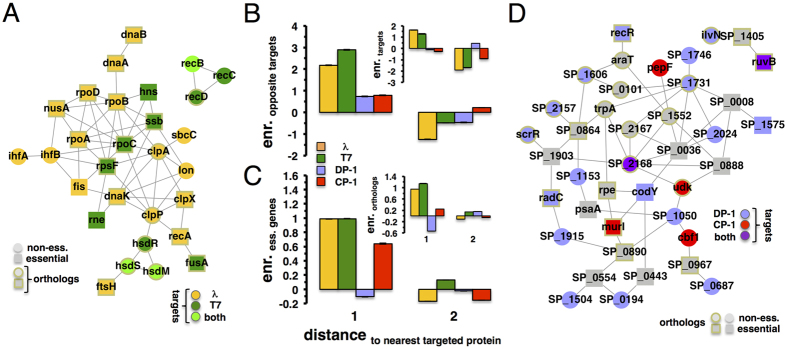
Clustering characteristics of targeted proteins. (**A**) Interactions between *E. coli* proteins that were targeted by bacteriophages lambda and T7 formed a large connected component (P < 10^−4^), capturing 21 out of 27 (77.8%) lambda targets and 11 out of 16 (68.8%) T7 targets. In the inset of (**B**) we grouped proteins in bins of the shortest distance to the nearest targeted protein in the underlying protein-protein interaction networks of *E. coli* and *S. pneumoniae*. In each distance bin, we calculated the enrichment of targeted proteins compared to a null model, randomly sampling sets of phage-specific targets. In contrast to targets of phages Dp-1 and Cp-1, targets of lambda and T7 are placed in the immediate vicinity of each other. Considering targets of T7, we observed that such proteins cluster in the immediate vicinity of lambda targets and *vice versa*, results that we obtained with targets of phages Dp-1 and Cp-1 as well. (**C**) Analogously, we observed that essential genes in *E. coli* strongly cluster around phage targets of lambda and T7. In turn, we found similar results for essential proteins in *S. pneumoniae* that were topologically located near targets of Cp-1 but not Dp-1. Furthermore, orthologous proteins clustered in the vicinity of phage targets (inset). Error bars indicate 95% confidence intervals. In (**D**), we mapped all interactions between proteins that were targeted by bacteriophages Dp-1 and Cp-1. We further considered all interactions involving essential or orthologous genes in *S. pneumoniae* that connected targeted proteins. Notably, we observed that such a network featured a significantly large connected component (P < 10^−4^), capturing 19 out of 28 (67.8%) Dp-1 targets and 6 out of 10 (60%) Cp-1 targets.

As for further clustering characteristics, we mapped all interactions between *E. coli* proteins that were targeted by bacteriophages lambda or T7 (Fig. 2A). Notably, we found a subnetwork that captured 21 out of 27 (77.8%) lambda targets and 11 out of 16 (68.8%) T7 targets. Qualitatively, such a network appeared to significantly pool essential *E. coli* genes and genes that have orthologs in *S. pneumoniae*. Applying Fisher’s exact test, we indeed found that targets of bacteriophages lambda and T7 are significantly enriched with essential (P < 10^−4^) and orthologous genes (P < 10^−7^). As for bacteriophages of *S. pneumoniae*, their targets failed to form a large connected component (data not shown) but seem to significantly accumulate proteins that have an ortholog in *E. coli* (P < 0.05) and essential genes (P < 0.15). To determine their tendency to cluster in the vicinity of targeted genes of the same bacteriophage, we grouped host proteins that were placed a given distance away from the nearest targeted proteins in the underlying protein-protein interaction network of *E. coli*. In each distance bin, we calculated the enrichment of targeted proteins compared to a null model where we randomly sampled sets of targeted proteins. The inset of Fig. 2B indicates that proteins that were targeted by lambda or T7 are placed in the network neighborhood of other proteins that were targeted by the same phage. Analogously, we determined the enrichment of bacteriophage targets of host proteins in *S. pneumoniae*, showing that Dp-1 and Cp-1 targets failed to cluster in close network vicinity of their corresponding targets. Although overlaps of target sets of organism-specific bacteriophages are limited, we investigated if such clustering characteristics can be extended when we considered the shortest distance to targets of the opposite phage. Surprisingly, the main plot of Fig. 2B suggests that targets of bacteriophage T7 were located in surprisingly close proximity to targets of lambda and *vice versa*, a result that held for targets of Dp-1 and Cp-1 as well (main plot, Fig. 2B). Previous analyses indicated that interactions between essential genes in *E. coli* were mostly organized in a large connected component^25^. As for *E. coli*, we found a connected component of 398 essential proteins in the underlying protein interactions network. Randomly sampling sets of essential genes we confirmed this result as statistically significant (P < 10^−4^). Analogously, we observed a significant giant component that was composed of 78 essential genes in *S. pneumoniae* (P < 10^−4^), generally suggesting that essential proteins cluster tightly. Determining their enrichment in bins to the nearest targeted proteins in *E. coli*, we indeed found that essential proteins tend to cluster in close proximity to proteins that were targeted by lambda and T7 (Fig. 2C). We obtained similar results when we considered essential proteins in the vicinity of Cp-1 targets in the underlying protein-protein interaction network of *S. pneumoniae*. Utilizing 781 ortholog pairs of proteins in *E. coli* and *S. pneumoniae* we found that such proteins formed a large connected component that was composed of 489 proteins in the interaction network of *E. coli* (P < 10^−4^). Furthermore, orthologous proteins in *S. pneumoniae* formed a large connected component with 156 proteins as well (P < 10^−4^). To investigate their clustering tendency, we found that *E. coli* proteins with orthologs in *S. pneumoniae* tend to cluster in the immediate vicinity of proteins targeted by lambda or T7. While we observed similar results when we considered targets of Cp-1 in *S. pneumoniae* (Fig. 2C), targets of Dp-1 failed to show such a trend.

Such clustering characteristics strongly suggest that targeted, essential, and orthologous genes in host organisms may form large, tightly connected subnetworks. In Fig. 2D, we mapped all interactions between proteins of *S. pneumoniae* that were targeted by bacteriophages Dp-1 and Cp-1. We also accounted for interactions of essential and orthologous proteins that connected phage targets. Notably, we obtained a network that featured a large connected component with 19 out of 28 (67.8%) Dp-1 targets and 6 out of 10 (60.0%) Cp-1 targets (Fig. 2D). Members of these connected components mediated processes that determined success of viral integration. For example, the *E. coli* subnetwork contains proteases (ClpX/A/B, etc.), endonucleases (HsdR/M/S, RecA, etc.), and transcriptional regulators (RpoA/B/C, IhfA/B, etc.). Such proteins reflect the machinery lambda uses for protein processing and phage assembly as well as gene regulation, given that it is a lysogenic phage. *S. pneumoniae*’s phages did not appear to use similar host activities, as patterns in target function are currently difficult to recognize.

**Figure 3:**
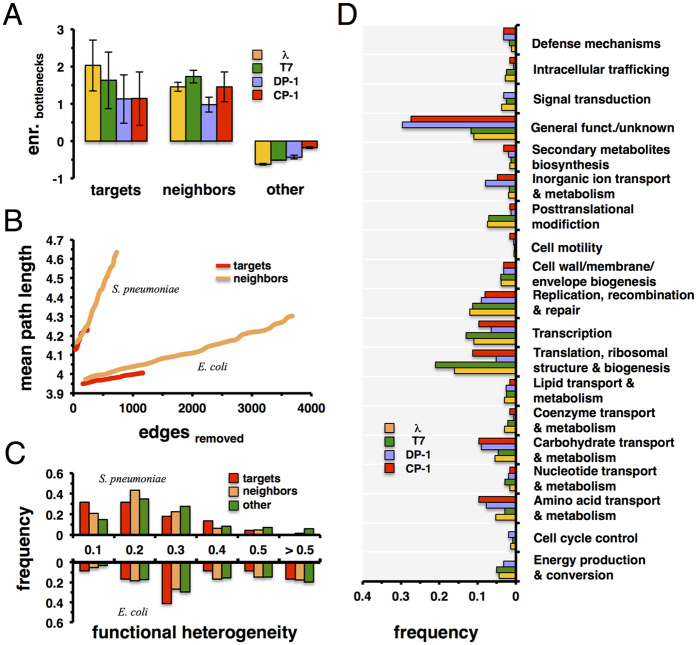
Nearest neighbors of targeted proteins. (**A**) We defined bottlenecks in the protein interaction networks of *E. coli* and *S. pneumoniae* as the top 20% of proteins with highest betweeness centrality. Furthermore, we determined their enrichment in sets of targeted proteins, their immediate neighbors, and remaining proteins. In general, bottlenecks are strongly enriched in sets of phage targets and proteins in their immediate vicinity, while they appear diluted in sets of remaining proteins. (**B**) To measure a protein’s impact on an interaction network’s resilience, we sorted all targeted proteins according to their degree in the interaction networks. We gradually deleted proteins and calculated the mean path length of the remaining proteins in the underlying interaction network. Analogously, we investigated the impact of a set of neighboring proteins of equal size, allowing us to observe that neighboring proteins had a higher disrupting impact on the networks topology than their corresponding targets. In (**C**) we determined all protein complexes in which bacteria-specific phage targeted proteins are involved. In particular, we calculated the functional heterogeneity of each protein complex. Furthermore, we determined analogous distributions when we considered complexes that involved neighbors of targeted and remaining proteins. In both organisms we observed that complexes that involved targets and their neighbors have higher functional heterogeneity than remaining clusters. (**D**) Focusing on targeted proteins, we identified all proteins in their immediate vicinity in the underlying protein-protein interaction networks of *E. coli* and *S. pneumoniae*. Based on the combined set of targeted and neighboring proteins we determined the frequency of such proteins that belong to the underlying functional classes.

The observed tendency of targeted, essential, and orthologous proteins to cluster in close proximity of other phage targets led us to hypothesize that proteins in the immediate vicinity of phage targets may carry global impact. Calculating the betweeness centrality of proteins in the underlying interaction networks of *E. coli* and *S. pneumoniae,* we defined the top 20% most central proteins as “bottleneck” proteins. Focusing on proteins that were targeted by bacteriophages in each host, we observed that such sets of central proteins were enriched with targeted proteins, compared to a null-model where we randomly sampled sets of bottleneck proteins (Fig. 3A). Focusing on the immediate neighbors of targeted proteins we observed that such proteins were enriched in sets of bottleneck proteins as well (Fig. 3A). In turn, we also considered remaining proteins that we found diluted in sets of bottleneck proteins (Fig. 3A). To measure a protein’s impact on an interaction network’s resilience, we performed a robustness analysis. We sorted all targeted proteins of bacteriophages Dp-1 and Cp-1 according to their degree in the underlying interaction network. Starting with the most connected protein we gradually deleted proteins and calculated the mean path length of the remaining protein interaction network after each deletion step. In comparison, we considered sets of equal size of proteins that interact with targeted proteins. Figure 3B indicates that the successive deletion of neighboring proteins had a higher impact on network topology by removing more edges that resulted in a higher mean path length. Notably, such observations held for *E. coli* phages as well.

In Fig. 3C, we focused on protein complexes that involved proteins that were targeted, neighboring, and remaining proteins. Considering the functional classes of proteins, we determined the functional heterogeneity of each protein complex defined as the Simpson diversity index^26^. Specifically, such a measure tends towards 1 if functions of proteins are similar and *vice versa*. In both organisms we observed that the distribution of complexes that involved targets and their neighbors were shifted to lower values, suggesting that targets and their neighbors secure a broad reach into different functions. In Fig. 3D, we determined the frequency of functional classes of proteins that are targeted and occur in their immediate vicinity. Compared to the distribution Fig. 1C indicates that such sets of proteins enforce the presence of transcription, replication, recombination, and repair functions while broadening the spectrum to other functions.

## Discussion

### Characteristics of bacteriophage-host interfaces

Determining interactions between proteins of bacteriophages Dp-1 and Cp-1 and their host *S. pneumoniae*, we compared their interaction patterns to corresponding observations in the interaction interface of bacteriophages lambda and T7 and their host *E. coli*. Although the phages are biologically different, we found that Cp-1 and Dp-1 share similarities with lambda and T7. In particular, we observed that all phages tend to target highly connected host proteins, have shorter paths to other non-targeted proteins, and connect protein complexes through the interactions of their targets. Furthermore, we observed that targets are enriched in bottleneck proteins, reiterating observations that hold true for human viruses^20^,^21^,^22^,^27^,^28^,^29^,^30^,^31^,^32^.

In turn, we observed that *E. coli* targets of bacteriophages lambda and T7 tend to cluster in close proximity to each other. Furthermore, we found that *E. coli* proteins with orthologs in *S. pneumoniae* and essential genes appear to cluster around phage-targeted proteins as well. Targets of bacteriophages in *E. coli* appeared to be strongly interconnected based on their network path lengths. In comparison, we found mixed clustering patterns characteristics when we considered targets of bacteriophages Dp-1 and Cp-1 and orthologous and essential proteins in *S. pneumoniae*. Our result may reflect the different ways that interactions between phages and host proteins have been determined. In particular, we collected interactions of phages lambda and T7 from many different sources that focused on the experimental determination of single interactions. In turn, we determined interactions of Dp-1 and Cp-1 on a large scale. Assuming that high-throughput approaches suffer from increased false negative rates, potential targets in the immediate neighborhood of proteins that interact with phages may have been missed. As a consequence, experimental focus on potential interactions that involve neighboring proteins may provide similar characteristics compared to the host-phage interactome of lambda and T7.

Although their targets hardly interconnect, we observed that orthologous proteins and essential genes of *S. pneumoniae* in the immediate vicinity of Dp-1 and Cp-1 targets allowed these proteins to organize in a large subnetwork. Such observations suggest that essential and conserved proteins may represent (in)direct gateways to take control of the underlying host cell. The role of immediate neighbors of bacteriophage targets in both hosts is further emphasized by their enrichment with bottleneck nodes and functional classes that are similar to phage targets and their functional heterogeneity. Such observations suggest that phages in general not only target responsive candidate genes to influence, but create a host-pathogen interface that appears confined to immediate network neighbors of targets in the underlying host protein interactions networks. Although such ‘extended’ host-phage interaction interfaces appear to have limited topological reach, phages manage to achieve global impact that permits the pathogens to quickly take control of the underlying host cell by reaching into various cellular functions.

### Phage biology and evolution

Differences between interaction patterns reflect differences in biology. Phages are exquisitely adapted to their hosts and exploit the resources their hosts provide. As a consequence, all phages are adapted to their host’s proteomes and interactomes. At this point hardly any detailed comparisons between phage and their relationships to their hosts exist, a surprise given that phages are among the fastest evolving species on earth. Furthermore, they represent an excellent model for genome, proteome, and interactome evolution. More data will be required to understand the dynamic processes involved in phage-host co-evolution.

### Protein function

Poorly annotated phage genomes pose another confounding problem in understanding phage biology. While lambda and T7 are well understood, Cp-1 and Dp-1 have only 12/28 (42%) and 44/77 (61%) of their proteins functionally annotated^12^,^13^. Furthermore, estimates suggest that there are tens if not hundreds of different phages per bacterial species^33^, implying that hundreds of unknown phage proteins interact with their hosts. The recent discovery of the phage CRISPR-Cas9 system^34^ has impressively shown that a large number of useful activities in phage proteomes exists that may also be used for phage therapy or other applications. We are convinced that PPIs will be a useful tool to investigate and illuminate these functions.

## Materials and Methods

### Molecular interactions data of *E. coli* and its phage

We collected 2,186 binary-Y2H interactions between 1,264 proteins in *E. coli* that were experimentally determined using a yeast-two-hybrid approach (Y2H) by Rajagopala *et al*.^2^. Furthermore, we utilized a total of 9,399 co-complex interactions between 2,044 proteins that were experimentally derived from large-scale tandem affinity purification approaches followed by mass spectrometry (AP/MS)^17^,^18^. Finally, we obtained 1,929 literature-curated binary interactions between 1,399 proteins^2^ that were largely curated from small-scale studies obtained by a multitude of methods. We collected 36 protein-protein interactions between 16 lambda and 23 *E. coli* proteins as well as 19 interactions between 8 T7 and 14 *E. coli* proteins from the literature^8^.

### Essential Genes

We used 712 essential proteins in *E. coli* as well as 436 essential genes in *S. pneumoniae* from the database of essential genes DEG10, an update of the database of essential genes (DEG) that collects data about essential genes from the literature^35^.

### Yeast two-hybrid screens of phage-host interactions

Proteins of Cp-1 (Uniprot proteome: UP000009089) and Dp-1 (UP000008920) were derived from previous studies^12^,^13^. These baits were cloned into pDEST32 and screened against a yeast two-hybrid (Y2H) array of 1,704 *S. pneumoniae* TIGR4 ORFs cloned into prey vector pDEST22 as described^7^. The strength of Y2H interactions was determined by increasing 3-amino-triazole (3-AT) concentrations up to 50 mM^36^. Tables 1 and 2 indicate 3AT scores, defined as 3AT_max_-3AT_background_. Specifically, 3AT_max_ is the maximal 3-AT concentration where a positive signal was found while 3AT_background_ is the 3-AT concentration where self-activation was suppressed.

### LuMPIS assays of phage-host PPIs

LuMPIS (Luminescence-based MBP pull-down Interaction screening system) assays were used to verify phage-host PPIs detected in the Y2H screens. Specifically, we used Gateway-compatible LuMPIS vectors with N-terminally MBP-tagged baits (in pCR3.1-N-MBP) to co-purify N-terminally eGFP-luciferase-tagged preys (in pCR3.1-N-eGFPLuc) in a pulled down assay via amylose beads. Proteins were expressed in human embryonic kidney cells (HEK) and raw protein extracts were used for the assay as described^37^,^38^. The pulled down preys were detected by measurement of the luciferase activity. Each PPI was measured as quadruplicates and compared to a quadruplicate negative control. The empty bait plasmid (MBP w/o ORF) was used in combination with the GFPluc preys to determine prey binding to MBP. PPIs with a luminescence intensity ratio (LIR) >3 were considered as positive.

### Protein complexes in *E. coli* and *S. pneumoniae*

We utilized a set of 517 protein complexes from a co-affinity purification study that was followed by mass spectrometry analyses^17^ in *E. coli*. We determined network clusters in the underlying protein interaction network of *S. pneumoniae* by utilizing the MCL algorithm^39^. In particular, we determined sets of clusters with a gradually increasing inflation parameter. Utilizing COG^15^,^16^ annotations, we calculated the functional coherence *fc* of cluster *i* as *fc*_*i*_* = fp*_*i*_*/p*_*i*_ where *fp*_*i*_ is the number of protein pairs that share a functional annotation, and *p*_*i*_ is the total number of annotated pairs in cluster *i*. Such a measure tends to increase with small clusters but decreases when more proteins are added. To balance such a trend, one maximizes the size of the given clusters by defining the modularity efficiency *E*_*M*_ as 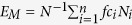, where *n* is the number of clusters, *N* is the total number of proteins while *N*_*i*_ is the number of proteins in the *i*th cluster^24^. In particular, we find a maximum of *E*_*M*_ when we used an inflation parameter of 1.6 in the case of *S. pneumoniae*, allowing us to obtain 148 clusters.

### Functional heterogeneity of protein complexes

Utilizing *N* classes of proteins that appear in a protein complex *i*, we calculated its functional heterogeneity as a Simpson diversity^26^ index defined as 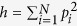, where *p*_*i*_ is the fraction of proteins of function *i*. Such a measure tends to 1 if proteins functions are similar.

### Protein Complex Participation Coefficient

For each protein that is part of at least one protein complex, we defined the protein complex participation coefficient of a protein *i* as 
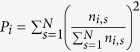
where *n*_*i,s*_ is the number of links protein *i* has to proteins in complex *s* out of *N* total complexes. If a protein predominantly interacts with partners of the same complex, *P* tends to 1^20^.

### Functional classes of proteins

*E. coli* and *S. pneumoniae* proteins were grouped according to broad functional classes that were defined by clusters of orthologous groups (COGs)^15^,^16^ since COGs provide a consistent classification of bacterial and eukaryotic species based on orthologous groups.

### Enrichment Analysis

Binning proteins with a certain characteristic *d* (e.g. with a given number of interactions) we calculated the fraction of proteins that had a feature *i* in each group *d, f*_*i*_*(d)*. As a null model we randomly sampled protein sets with feature *i* of the same size 10,000 times and calculated the corresponding random fraction, *f*_*i,r*_ (*d*). The enrichment/depletion of proteins with feature *i* in a group *d* is then defined as 
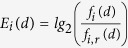
.

### Enrichment analysis as a function of degree

We grouped phage proteins according to their number of interactions in an underlying bacterial protein interaction network. We represented each group by *N*_≥*k*_ proteins that had at least *k* interactions and calculated the number of targeted proteins *i, N*_*i*,≥*k*_ in each group. Randomly picking targeted genes we defined 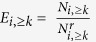
as their enrichment where 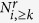 was the corresponding random number of targeted proteins among all *N*_i,≥*k*_ proteins. After averaging *E*_*i*_ over 10,000 randomizations *E*_*i*_ > *1* pointed to an enrichment and *vice versa*, while *E*_*i*_ ~ *1* indicated a random process^40^.

### Orthologous proteins

Utilizing all-versus-all BLASTP searches determined by the InParanoid script^41^ in protein sets of *E. coli* and *S. pneumoniae*, sequence pairs with mutually best scores were selected as central orthologous pairs. To enhance quality, we only accepted BLAST matches with a score >40 bits, covering at least 50% of the longer sequence. Proteins of both species that showed such an elevated degree of homology were clustered around these central pairs, forming orthologous groups. The quality of the clustering was further assessed by a standard bootstrap procedure. We only considered the central orthologous sequence pair that provided a confidence level of 100% as the real orthologous relationship, allowing us to obtain 781 orthologous protein pairs between *E. coli* and *S. pneumoniae.*

### Bottleneck proteins

As a global measure of its centrality, we defined betweeness centrality *c*_*B*_ of a protein *v* as 
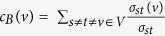
 where *σ*_*st*_ was the number of shortest paths between proteins *s* and *t* while *σ*_*st*_ (*v*) was the number of shortest paths running through protein *v*. As a set of bottleneck proteins we defined the top 20% of proteins with highest betweeness centrality.

## Additional Information

**Data availability**: The protein interactions from this publication have been submitted to the IMEx (http://www. imexconsortium.org) consortium through IntAct (ref. 42) and assigned the identifier IM-25020.

**How to cite this article**: Mariano, R. *et al*. The interactome of *Streptococcus pneumoniae* and its bacteriophages show highly specific patterns of interactions among bacteria and their phages. *Sci. Rep.* **6**, 24597; doi: 10.1038/srep24597 (2016).

## Figures and Tables

**Table 1:** Interactions between proteins of *S. pneumoniae* and its phage Cp-1.

**Phage protein**		**Host protein**		**3AT**	**LIR**
Cpl1	Lysozyme	SP_1208	uridine kinase	47.5	4
orf6	Hypothetical protein	SP_1713	transcriptional regulator, NrdR family	50	6
orf10	Connector protein	SP_1354	ribosomal protein L7/L12	2.5	12
orf10	Connector protein	SP_1881	glutamate racemase	50	6
orf16	Hypothetical protein	SP_0259	Holliday junction DNA helicase RuvB	1	12
orf17	Tail protein N	SP_0979	oligoendopeptidase F	50	14
orfb	Hypothetical protein	SP_2168	putative fucose operon repressor	2.5	1
orfb	Hypothetical protein	SP_0859	membrane protein	25	1
orfb	Hypothetical protein	SP_1980	cmp-binding-factor 1	25	2
orfb	Hypothetical protein	SP_1213	conserved domain protein	50	240
orfc	Hypothetical protein	SP_0979	oligoendopeptidase F	2	3

Host proteins are given as locus numbers. 3AT is the highest 3-AT concentration at which this interaction was detected. LIR are luminescence intensity ratios from LuMPIS assays rounded to the nearest integer (see text for details). Combinations of high 3AT score and LIR values are most reliable.

**Table 2:** Interactions between proteins of *S. pneumoniae* and its phage Dp-1.

**Phage protein**		**Host protein**		**3AT**	**LIR**
orf4	Queuosine biosynth. protein QueE	SP_2036	PTS system, IIA component	25	454
orf9	No similarity	SP_1504	TPR domain protein	0.5	19
orf9	No similarity	SP_0259	Holliday junction DNA helicase RuvB	50	11
orf9	No similarity	SP_1395	putative phosphate transport system regulatory protein PhoU	50	23
orf9	No similarity	SP_2168	putative fucose operon repressor	50	15
orf12	Holliday junction resolvase RecU	SP_2168	putative fucose operon repressor	0.1	2
orf14	dUTPase	SP_2125	conserved hypothetical protein	50	45
orf16	NAD-dependent DNA ligase	SP_0259	Holliday junction DNA helicase RuvB	50	5
orf18	DNA polymerase III, delta’ subunit HolB	SP_1584	GTP-sensing transcriptional pleiotropic repressor CodY	24.5	6
orf29	Hypothetical protein	SP_2012	glyceraldehyde 3-phosphate dehydrogenase	0.25	3
orf31	Hypothetical protein	SP_2168	putative fucose operon repressor	0.2	5
orf31	Hypothetical protein	SP_1153	hypothetical protein	0.25	6
orf31	Hypothetical protein	SP_0259	Holliday junction DNA helicase RuvB	0.5	4
orf32	Hypothetical protein	SP_0194	conserved hypothetical protein	47.5	5
orf32	Hypothetical protein	SP_0259	Holliday junction DNA helicase RuvB	47.5	3
orf32	Hypothetical protein	SP_1540	single-strand binding protein *Ssb*	47.5	3
orf32	Hypothetical protein	SP_1669	MutT/nudix family protein	47.5	10
orf32	Hypothetical protein	SP_1915	hypothetical protein	47.5	5
orf33	Hypothetical protein	SP_1088	DNA repair protein RadC	0.5	3
orf34	Hypothetical protein	SP_2157	alcohol dehydrogenase, iron-containing	2	35
orf34	Hypothetical protein	SP_0446	acetolactate synthase, small subunit	9	12
orf39	Zinc finger protein	SP_0259	Holliday junction DNA helicase RuvB	50	10
orf44	Rho-like domain lipoprotein	SP_1725	sucrose operon repressor	2.25	3
orf44	Rho-like domain lipoprotein	SP_2157	alcohol dehydrogenase, iron-containing	2.25	13
orf44	Rho-like domain lipoprotein	SP_1050	putative transcriptional regulator	4.75	2
orf44	Rho-like domain lipoprotein	SP_1536	conserved hypothetical protein	4.75	10
orf44	Rho-like domain lipoprotein	SP_0446	acetolactate synthase, small subunit	9.75	6
orf44	Rho-like domain lipoprotein	SP_1575	conserved hypothetical protein	9.75	14
orf47	Hypothetical protein	SP_0687	ABC transporter, ATP-binding protein	49	4
orf48	Hypothetical protein	SP_2168	putative fucose operon repressor	0.5	3
orf48	Hypothetical protein	SP_1746	conserved hypothetical protein	2.5	3
orf51	Hypothetical protein	SP_1672	recombination protein RecR	0.5	6
orf58	Holin	SP_1505	membrane protein	1	1
orf58	Holin	SP_1731	conserved hypothetical protein	2.5	3
orf58	Holin	SP_1606	glycosyl transferase, family 2	25	3
orf58	Holin	SP_1751	putative transporter, CorA family	25	5
orf60	Hypothetical protein	SP_2024	PTS system, IIA component	10	5
orf72	Membrane protein	SP_1606	glycosyl transferase, family 2	50	50

Host proteins are given as locus numbers. 3AT is the highest 3-AT concentration at which this interaction was detected. LIR are luminescence intensity ratios from LuMPIS assays rounded to the nearest integer (see text for details). Combinations of high 3AT score and LIR values are most reliable.
